# Developing an assessment workflow for 505(b)(2) products within the outpatient setting within a large academic medical center

**DOI:** 10.1093/ajhp/zxag063

**Published:** 2026-03-06

**Authors:** Chloe Matsuda, Russell Findlay, Erin Fox

**Affiliations:** University of Utah Health Pharmacy Services, Salt Lake City, UT, USA; University of Utah Health Pharmacy Services, Salt Lake City, UT, USA; University of Utah Health Pharmacy Services, Salt Lake City, UT, USA

**Keywords:** 505(b)(2) products, HCPCS code, therapeutic equivalence, drug approval, united states food and drug administration, drug policy

## Abstract

**Purpose:**

To describe the development of a proactive workflow to identify medications approved through the 505(b)(2) pathway and anticipate potential challenges they may pose to the medication-use process within a large multisite health system.

**Summary:**

The baseline workflow was based on the health system’s response to a potential mix-up with bendamustine products, which resulted in the formation of a 505(b)(2) task force. An Excel tool was created to visualize and track changes to Centers for Medicare and Medicaid Services (CMS) ASP NDC-HCPCS crosswalk files. A 505(b)(2) directory was created to store the changes identified from the Excel tool. A list of 505(b)(2) medications was assessed with a prioritization matrix and gap analysis. The baseline workflow and gap analysis informed the creation of a new proactive workflow. The top-priority medication identified from the prioritization matrix was selected to trial the new workflow.

**Conclusion:**

A proactive, interdisciplinary approach is essential to effectively manage the evolving challenges associated with 505(b)(2) medications. Implementing structured tools, including a crosswalk comparison tool, directory, and prioritization matrix, provided a systematic method to identify and monitor these products within a complex regulatory environment. Continued refinement will be necessary as CMS and Food and Drug Administration policies evolve, but this model offers a framework that may be adapted to different care settings.

Key Points

- The Hatch-Waxman amendments of 1984 introduced 505(b)(2) medications, but those medications now present distinct challenges after CMS’s decision to reclassify them as single-source products.
- Pharmacy teams must treat single-source 505(b)(2) products as stand-alone medications rather than grouping them with other generic drugs.
- Implementing a proactive system for monitoring ASP NDC-HCPCS crosswalks and establishing metrics to prioritize efforts can help institutions face the challenges these products pose.

There are 3 main drug approval pathways: the 505(b)(1) pathway, the 505(j) pathway, and the 505(b)(2) pathway. The 505(b)(1) pathway is the traditional new drug application (NDA) that requires the applicant to submit original data that demonstrates safety and efficacy. The NDA results in the approval of a new pharmaceutical.^1^ Therapeutic equivalence is not evaluated with the 505(b)(1) pathway because that pathway is for approval of the marketing and sale of a new, stand-alone drug. The Drug Price Competition and Patent Term Restoration Act of 1984 (also known as the Hatch-Waxman amendments) established the 505(b)(2) and 505(j) abbreviated pathways.^2^ The 505(j) pathway is commonly known as the abbreviated new drug application (ANDA) pathway. The ANDA results in the approval of a generic medication with data supporting bioequivalence to the reference listed drug (RLD).^1,3^ New studies for safety and efficacy are not required because an ANDA relies on existing data for the RLD.^1,3^ After the approval of an ANDA, therapeutic equivalence to the RLD is assigned and listed in the US Food and Drug Administration (FDA) Orange Book.^1,3^ Medications that are A rated in FDA’s Orange Book are considered therapeutically equivalent and can be interchanged.

The 505(b)(2) pathway is also an NDA pathway. However, unlike the 505(b)(1) pathway, the 505(b)(2) pathway results in the approval of a new chemical or molecular entity derived from small changes to a previously approved drug.^1,3^ Medications approved through the 505(b)(2) pathway are similar to an existing medication but can differ in characteristics like dosage form, strength, route of administration, formulation, excipients, dosing regimen, indication, active ingredient (different salts), preparation instructions, and stability. FDA allows these products to be labeled with identical names, despite the different clinical characteristics and risks for mix-ups. The 505(b)(2) pathway requires full reports of safety and efficacy. The applicant is permitted to use studies not conducted by or for the applicant and for which the applicant has not obtained a right or reference for use, such as previously published studies.^1,3^ After the approval of a 505(b)(2) medication, FDA may assign therapeutic equivalence; however, the evaluation is not automatic.^3^ 505(b)(2) medications are often confused with biosimilar medications and branded generics; however, they are all distinct products because biosimilars are approved through the 315(k) pathway and branded generics are approved through the 505(j) pathway. The 505(b)(2) and 505(j) pathways reduce development time and cost by allowing applicants to rely on existing studies instead of conducting original research, unlike the traditional NDA route. Since 2015, approximately 50% of NDAs submitted each year have gone through the 505(b)(2) pathway.^4^

The 505(b)(2) pathway is not new, but it introduced significant challenges after the Centers for Medicare and Medicaid Services (CMS) started considering each product to be from a single source. Prior to 2023, CMS commonly assigned 505(b)(2) products the same Healthcare Common Procedure Coding System (HCPCS) code as the reference drug and its generics.^4^ However, CMS decided that effective January 1, 2023, it would consider 505(b)(2) medications as single-source products if they were not rated by FDA in the Orange Book as therapeutically equivalent to any other drug.^5^ Most 505(b)(2) drugs are not evaluated for therapeutic equivalence and are therefore considered single-source products. For 505(b)(2) medications approved after January 1, 2023, CMS assigned a unique HCPCS code based on the specific manufacturer’s average sales price (ASP).^4^ Additionally, CMS went back and reassigned HCPCS codes to previously approved 505(b)(2) products that met the definition of single-source.^4^ On rare occasions, 505(b)(2) medications are reviewed for and receive therapeutic equivalence, so they are considered multiple-source products and share the same HCPCS code as the medication they are equivalent to.^4^ CMS recommends that providers use the ASP files, specifically the NDC-HCPCS crosswalk files, to determine the most appropriate HCPCS code to use on claims.^5^

## Problem

The management of 505(b)(2) medications presents significant challenges due to their regulatory complexity, lack of standardized identification, and frequent misclassification within drug databases and purchasing systems. Unlike medications approved through the ANDA pathway, 505(b)(2) medications with unique HCPCS codes must be treated as separate entities and cannot be interchanged with similar drugs even though they may have a similar role in treatment. 505(b)(2) medications are hard to identify without looking at the FDA product approval letter because neither CMS nor FDA maintain a centralized database. CMS encourages providers to use the quarterly ASP NDC-HCPCS crosswalk to identify the correct billing code; however, it does not list the approval pathway or therapeutic equivalence ratings. It is crucial that the pharmacy team identify and use the correct billing code for each medication to ensure reimbursement from insurance companies. Another challenge is that many drug databases may incorrectly assume 505(b)(2) products are interchangeable, so they consolidate them into a single medication record with medications that have the same route, dosage form, dose, and active ingredient.^4^ When the database sends the file to the pharmacy information system, all drugs within a single medication record are treated as equivalent, even though single-source 505(b)(2) products should be listed as distinct entries, and the products may have very different clinical characteristics.^4^ This may result in errors in purchasing, product selection, substitution, administration, electronic health record (EHR) builds, billing, and reimbursement.^4^ Additionally, 505(b)(2) products have a similar naming convention to generics and are often inappropriately labeled in purchasing portals, which results in buyers mistakenly purchasing a 505(b)(2) product, thinking it is a generic medication.^4^ To add further confusion, 505(b)(2) products may have a “brand” name. 505(b)(2) products are also subject to ongoing changes as newly approved drugs receive unique HCPCS codes, as CMS reassigns previous approvals that meet the definition of single-source, and as FDA assignment of therapeutic equivalence shifts a drug from single-source status (ie, a product with a unique HCPCS code) to multiple-source status (ie, a product with a shared HCPCS code).^4^ Pharmacies must develop a method to identify and separate 505(b)(2) productsm, and the evolving regulatory landscape requires a high level of collaboration to ensure the safe and correct use of 505(b)(2) products.

## Background

The University of Utah is an academic medical center located in Salt Lake City that encompasses 5 hospitals and 17 community health centers. We have 4 infusion centers located across the Salt Lake Valley and a home infusion service. Our system has over 4 million patient visits annually and serves patients across the Mountain West region. The pharmacy purchasing team oversees the purchasing of all medications for inpatient locations, outpatient clinics, and infusion centers.

The purchasing team had assigned Bendeka as the primary bendamustine product when a potential mix-up occurred. Our drug database listed a bendamustine hydrochloride product as a generic equivalent, so it was purchased mistakenly. A technician was preparing a dose of bendamustine and noticed that the generic bendamustine hydrochloride vial they scanned looked different than the usual Bendeka vial. This prompted the technician to ask a pharmacist if the vial was equivalent to Bendeka. The pharmacist determined that Bendeka is a 505(b)(2) product and the concentration and infusion instructions were different from the generic bendamustine hydrochloride vial. Upon further investigation, the generic bendamustine hydrochloride was incorrectly listed as a generic equivalent in the wholesaler database, so it was considered to be equivalent to Bendeka despite having a different concentration and administration instructions. This near miss triggered a larger discussion within the pharmacy department about the absence of a workflow dedicated to identifying 505(b)(2) products. To address this gap, we set out to create a proactive process to identify and address 505(b)(2) products. Our project was focused on creating a workflow, and no new data were generated or analyzed in support of this article.

## Analysis and resolution

### Assembling the task force

To address the widespread impact of 505(b)(2) challenges across our teams and improve coordination among our many sites throughout the valley, we established a dedicated task force to centralize communication and streamline our efforts. Our taskforce consisted of key stakeholders including representatives from the areas of oncology, infusion, home infusion, information technology, 340B Drug Pricing Program participation, drug information, purchasing, and operations. The purpose of the task force was to assess current gaps and future needs related to 505(b)(2) medications and guide the development and implementation of a proactive workflow. Initially, the task force met virtually on an ad hoc basis for progress updates and to discuss key decisions. The goal behind an established task force was to avoid disruption to teams and create efficiency with a collaborative team rather than discussions with each different team on an as-needed basis.

The changes enacted by CMS impacted pharmacy departments nationwide and created unique challenges with 505(b)(2) products. CMS encouraged the use of the ASP NDC-HCPCS crosswalks to identify the appropriate billing code, but our institution did not have a defined process for using them. The crosswalks are published quarterly and include extensive details like billing code, short description, labeler name, NDC, drug name, HCPCS dosage, package size, package quantity, and billing units. While the crosswalks provide a high level of detail, they have several limitations, including the absence of labels or markers for 505(b)(2) products and the need to manually compare each quarterly file to detect updates. Additionally, there is often a lag between newly approved medications and the assignment of a designated HCPCS code.^6,7^

### Building on solutions from other institutions

**Figure 1.**
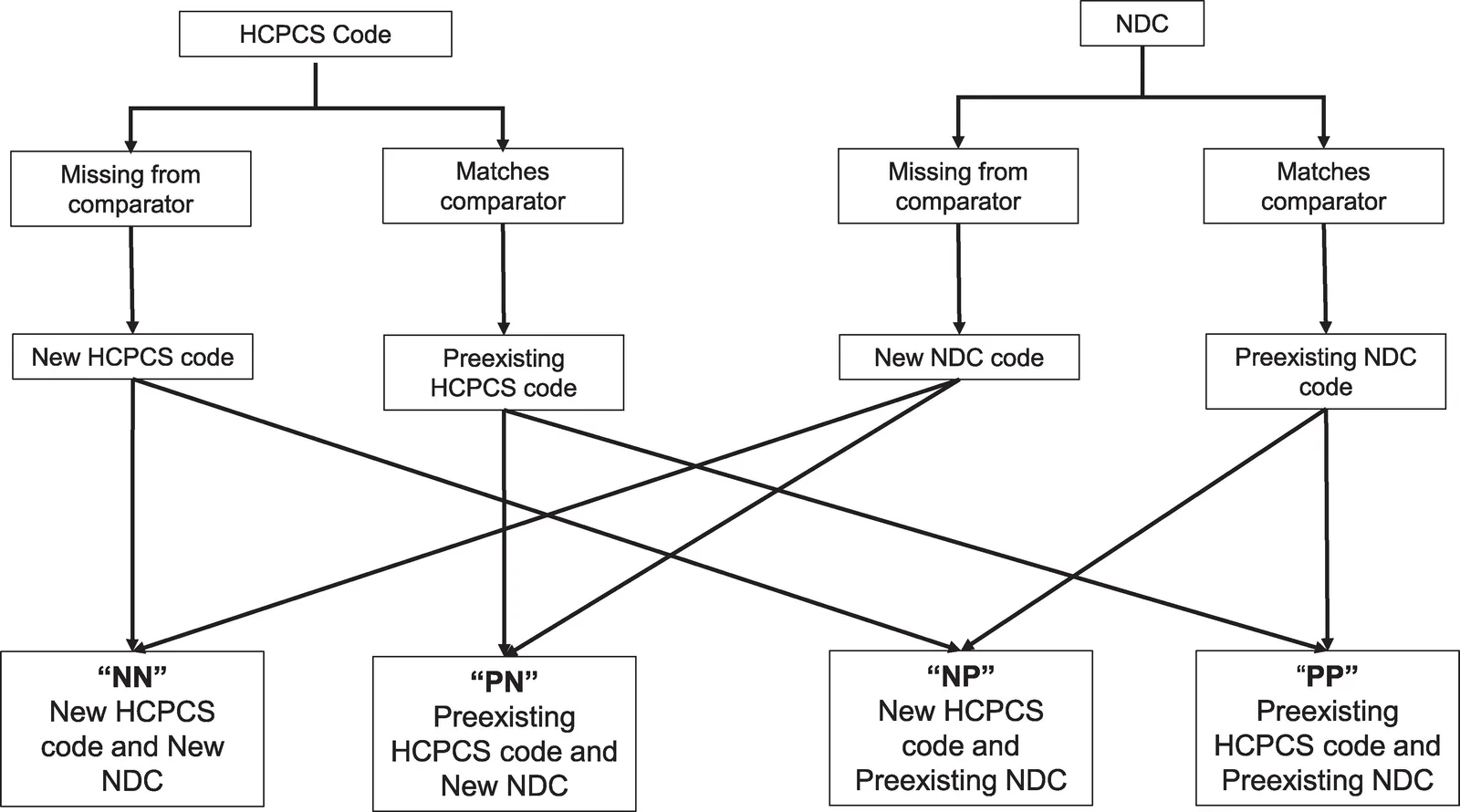
This flowchart illustrates the decision tree within the Microsoft Excel formula that determines whether a product in the average sales price (ASP) NDC-HCPCS crosswalk is classified as NN, NP, PN, or PP. HCPCS indicates Healthcare Common Procedure Coding System; NDC, National Drug Code.

Our institution did not have an efficient process for reviewing the crosswalks, so we expanded on ideas presented at informational sessions and built an Excel tool to easily compare ASP NDC-HCPCS crosswalks. After transcribing the crosswalks into the tool, sequenced application of Excel’s IFERROR and VLOOKUP functions are used to compare the crosswalk files and identify different combinations of HCPCPS codes and NDCs. The 4 combinations are as follows: new code and new NDC (NN), new code and preexisting NDC (NP), preexisting code and new NDC (PN), and preexisting code and preexisting NDC (PP). Figure 1 visualizes the logic behind the Excel formula.

When comparing the April 2025 and July 2025 quarterly ASP NDC-HCPCS crosswalks, there were 155 combinations of NN, 155 combinations of NP, 316 combinations of PN, and 7,247 combinations of PP. For example, HCPCS code J9292 and NDCs 83831-0131-01 and 83831-0132-01 were identified by our Excel tool as NN. This J code and NDCs are associated with pemetrexed dipotassium injection sold under the proprietary name Axtle. Upon further review, the approval letter posted on the Drugs@FDA database states that this pemetrexed for injection was submitted under NDA 210661 via the 505(b)(2) pathway. Axtle is approved for the same indications and is available in the same strength vials and the same dosage form as many of the ANDA pemetrexed products. However, it is not listed as A rated in FDA’s Orange Book, so it is considered by CMS as a single-source product and therefore cannot be used interchangeably. If our health system used Axtle rather than one of the ANDA pemetrexed products, there would be no clinical concerns, but there would be financial concerns as our organization may not receive payment.

We intentionally designed a user-friendly tool that only requires the user to copy and paste the crosswalks into the proper sheet. Users can sort the combinations of HCPCS codes and NDCs to focus on one group of combinations at a time to maximize efficiency. The most common combination is PP because the quarterly crosswalk updates have a small number of changes. The task force focuses most of their efforts on the combination of NN because it is the most likely to represent a newly approved 505(b)(2) product. The combination of NP could represent a product with a reassigned HCPCS code. The combination of PN could represent a product that is therapeutically equivalent to other medications. 505(b)(2) products identified from the crosswalk and tertiary resources are stored in the 505(b)(2) directory, which is a running Excel file that stores critical information, including the HCPCS code, application number, approval pathway, strength, dosage form, company, and therapeutic equivalents.

### Creating a prioritization matrix

From 2015 to 2023, FDA approved 509 505(b)(2) medications. The timing of the CMS decision to assign unique HCPCS codes did not allow our system to promptly evaluate all 505(b)(2) products due to the high volume, unique complexity of each product, and competing responsibilities. As a result, the task force identified the need to strategically focus efforts on medications that pose the most significant risk of operational, clinical, and financial challenges to ensure we direct resources where they will be most impactful.

**Figure 2.**
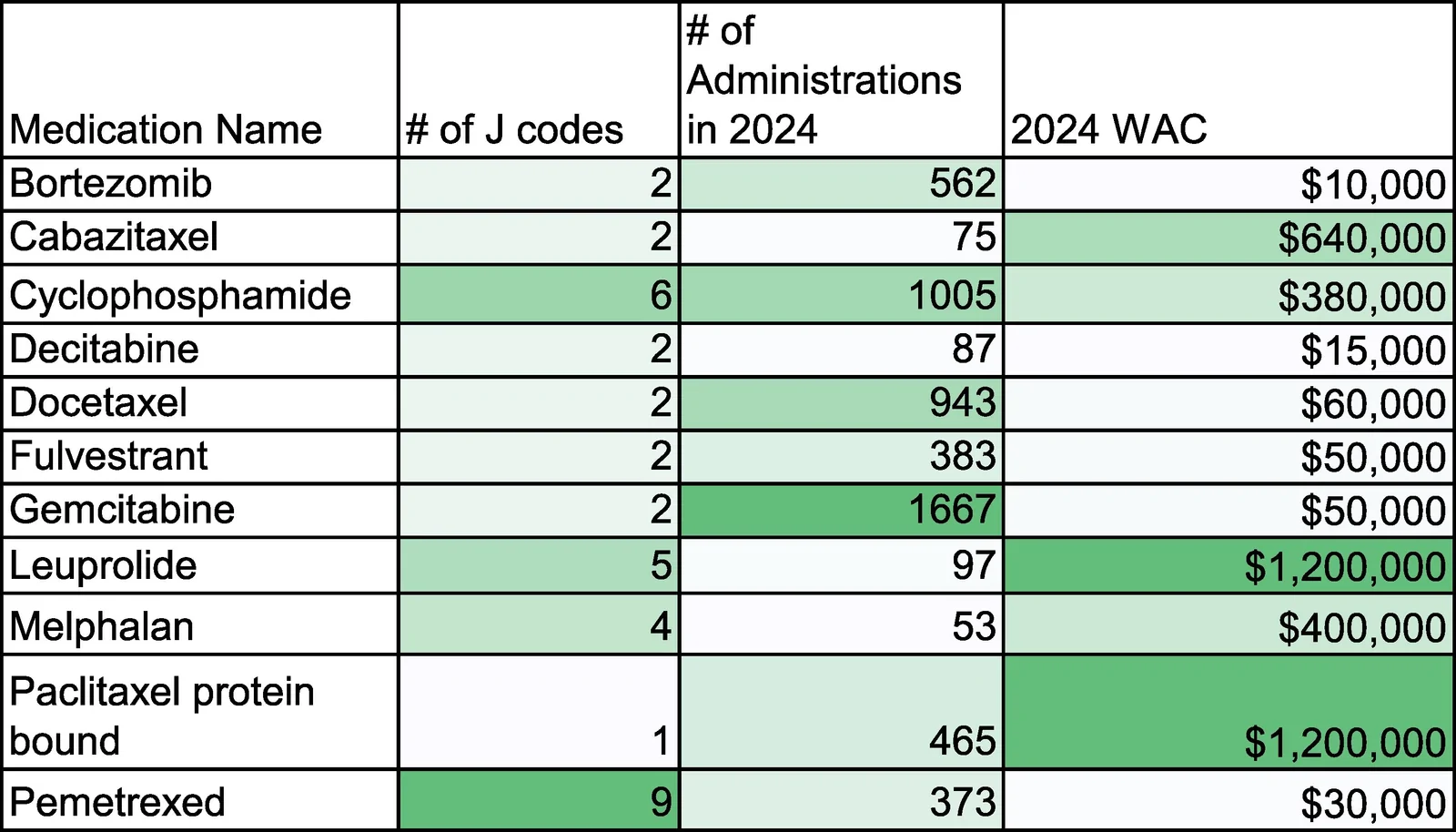
The prioritization matrix assesses 505(b)(2) medication by J code volume, administrations, and annual expense. Cyclophosphamide was identified as a high-priority medication due to its large number of J codes, administrations, and expense. WAC indicates wholesale acquisition cost.

We designed a prioritization matrix using the HCPCS code volume, usage, and cost to estimate how likely each medication is to present challenges. We measured HCPCS code volume by the number of active J codes, usage by the number of administrations within a calendar year, and cost by the annual expense. The task force agreed that the highest-risk medications requiring urgent discussion are those with a high HCPCS code volume, high usage, and high cost. However, more investigation is needed to validate and refine the assessments from the matrix. After designing the prioritization matrix, we collected the data for each metric for the 505(b)(2) medications our system commonly used in outpatient clinics and infusion services. Figure 2 shows an example of a prioritization matrix. The task force identified cyclophosphamide from the prioritization matrix, which prompted further investigation into our payers, purchase history, and primary product strategy.

After selecting cyclophosphamide, discussion amongst the task force revealed that historically, our institution used a powder for solution formulation but switched to solution a few years ago to optimize operational efficiency. After reviewing the 505(b)(2) directory, we determined that the 505(b)(2) cyclophosphamide products are IV solutions, and the generic (ANDA) cyclophosphamide products are IV powders for solution. The purchasing team informed the task force that our primary product is a 505(b)(2) IV solution. The oncology team communicated that only one payer had pushed back against our primary product, but that payer recently changed and now approved all HCPCS codes for cyclophosphamide. The information technology (IT) team informed the task force that cyclophosphamide IV solution is grouped under a single medication record. After all the representatives weighed in, the task force discussed switching back to the powder for solution formulation to avoid the 505(b)(2) solutions. However, since all our payers approve every HCPCS code for cyclophosphamide, the task force decided to continue with our current primary product rather than switching. The task force also decided not to pursue separate medication entries for each NDC of cyclophosphamide, as the time and resources required to build them manually outweighed the limited benefit.

### Implementing a new, proactive workflow

**Figure 3.**
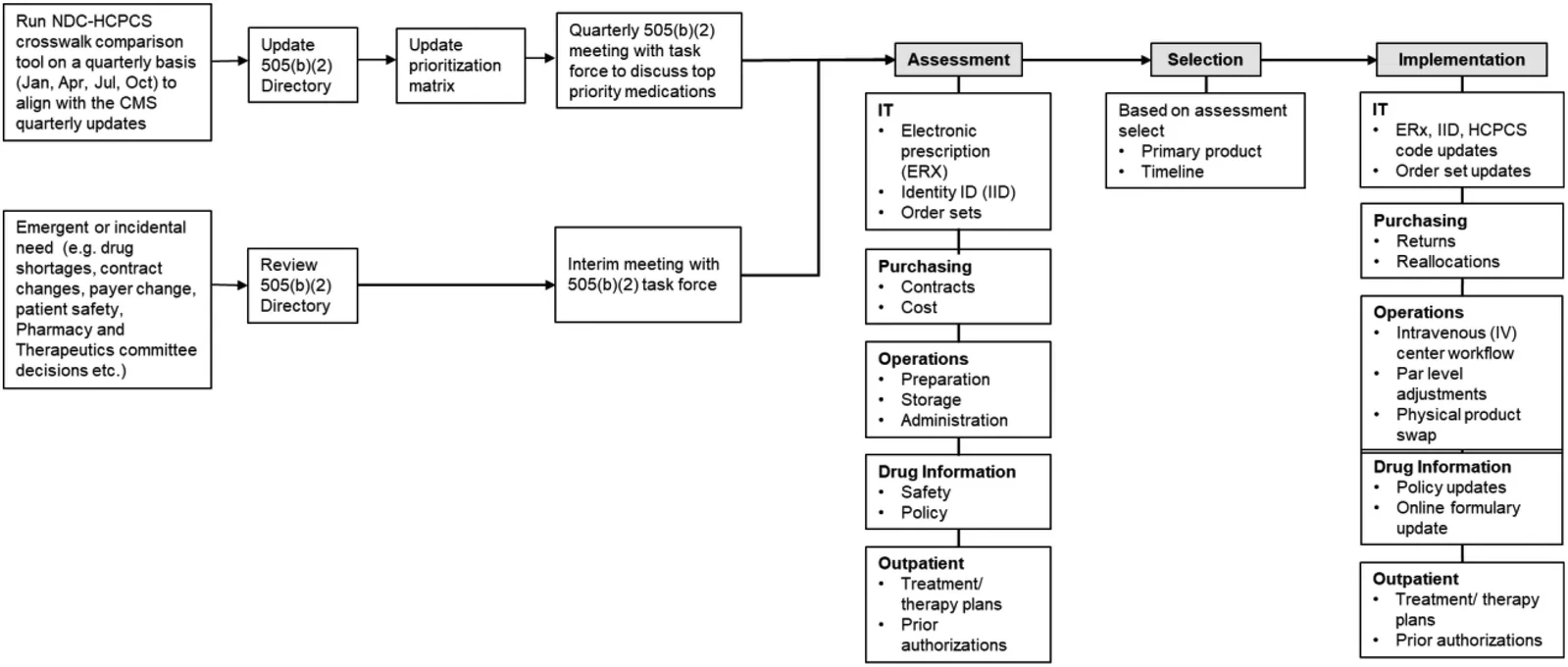
This flowchart outlines the structured process to assessment, decision-making, and implementation of interventions related to 505(b)(2) medications. The workflow is activated by regular review aligned with the Centers for Medicare and Medicaid Services (CMS) average sales price (ASP) NDC-HCPCS crosswalk updates and by emergent or incidental needs. HCPCS indicates Healthcare Common Procedure Coding System; NDC, National Drug Code.

The new workflow emphasizes proactivity through scheduled recurring task force meetings that align with CMS’s quarterly NDC-HCPCS crosswalk updates. Figure 3 showcases this new workflow. The goal of the quarterly meetings is for the task force to use the crosswalk comparison tool, directory, and prioritization matrix to assess potential problems and implement the necessary solutions to prevent those problems from coming to fruition. The new workflow also encompasses the unpredictable nature of the healthcare field by having interim task force meetings for incidental needs like drug shortages, contract changes, or safety events. When the task force meets to discuss 505(b)(2) medications, each stakeholder explains unique considerations to facilitate discussion about a primary product strategy. IT personnel discuss impacts to the EHR. Purchasing representatives highlight impacts on contracts, procurement cost, returns, and reallocations. Operations personnel explain the impacts on preparation, storage, administration, IV center workflow, periodic automatic replenishment level adjustments, and physical product swaps. Drug information specialists provide insights on formulary considerations, safety, and impact to internal policies. The oncology and infusion teams provide perspective on treatment and therapy plans, prior authorizations, and payer preferences.

## Discussion

Managing 505(b)(2) medications would not be required if FDA proactively assigned therapeutic equivalence ratings in the FDA Orange Book. CMS’s reclassification, with limited notification to implement solutions, sparked challenges with operations, purchasing, and reimbursement because medication information systems group 505(b)(2) products with similar medications in cases when CMS has determined they should be stand-alone products. Further, FDA approves 505(b)(2) medications that have significant clinical differences but allows them to have the same generic drug name without any further distinction. Forming a task force was the first step in creating a proactive approach to navigate the complexities of 505(b)(2) products. The crosswalk comparison tool and directory allowed staff members to efficiently identify 505(b)(2) products and provided a systematic approach to monitor the continuously evolving landscape proactively. One limitation of the crosswalk comparison tool is that it cannot explain why a medication was assigned to a specific J code and if the medication is therapeutically equivalent with other medications, which is why pharmacy staff members must do more investigation with references like Drugs@FDA website and the National Library of Medicine’s DailyMed website. Another limitation is that pharmacy staff must run the tool throughout the quarter to catch billing updates that occurred outside of the scheduled quarterly updates. The prioritization matrix provided an objective framework to rank medications based on the defined metrics to estimate the highest-risk medications. The new workflow incorporated routine reviews to ensure consistent surveillance of CMS NDC-HCPCS updates and incidental evaluations to ensure timely response to unexpected changes involving 505(b)(2) products. The proactive approach and tools optimized 505(b)(2) identification and informed decision-making regarding EHR builds, product selection, and reimbursements. These methods may be applied to other settings where 505(b)(2) products are used, including the inpatient setting. Continual improvement to the workflow and tools are needed as 505(b)(2) products evolve.

## Conclusion

A proactive, interdisciplinary approach is essential to effectively managing the complexities of 505(b)(2) medications. Implementing structured tools and workflows enables institutions to maintain compliance, optimize reimbursement, and ensure safe medication use amid ongoing regulatory changes from CMS and FDA.

## Data Availability

No new data were generated or analyzed in support of this work.
